# Evolution, reproduction and definition of life

**DOI:** 10.1007/s12064-013-0184-5

**Published:** 2013-05-15

**Authors:** Krzysztof Chodasewicz

**Affiliations:** College of Physiotherapy in Wrocław, Wrocław, Poland

**Keywords:** Definition of life, Darwinian definition, ‘Mule’s problem’, Life as a collective phenomenon

## Abstract

Synthetic theory of evolution is a superior integrative biological theory. Therefore, there is nothing surprising about the fact that multiple attempts of defining life are based on this theory. One of them even has a status of NASA’s working definition. According to this definition, ‘life is a self-sustained chemical system capable of undergoing Darwinian evolution’ Luisi (Orig Life Evol Bios 28:613–622, 1998); Cleland, Chyba (Orig Life Evol Bios 32:387–393, 2002). This definition is often considered as one of the more theoretically mature definitions of life. This Darwinian definition has nonetheless provoked a lot of criticism. One of the major arguments claims that this definition is wrong due to ‘mule’s problem’. Mules (and other infertile hybrids), despite being obviously living organisms, in the light of this definition are considered inanimate objects. It is strongly counterintuitive. The aim of this article was to demonstrate that this reasoning is false. In the later part of the text, I also discuss some other arguments against the Darwinian approach to defining life.

## Problems with the definition of life

Defining life is a non-standard practice for biologists. This is mainly due to the fact that biologists can make fruitful research without this definition (Emmeche 1992). It is even true in case of objects having an unclear status such as viruses. However, this is unequivocally true only in case of ‘standard’ biology. New biological fields of research such as astrobiology, the origins of life, artificial life, and synthetic biology seem to be strongly dependent on the definition of life. As suggested by Oliver and Perry, some authors even believe that the lack of the definition of life is necessary for further progress in these disciplines (2006). Let us consider astrobiology: if we want to project a space probe to search for life on other planets, we have to assume some kind of life definition which allows us to differentiate living things.

With regard to the origin of life, an interesting analysis has been made lately by Addy Pross. He argues that there are three major questions of modern biology: what is life?, how can we make a simple living object?, and how did life emerge? He also argues that these questions are strongly connected with each other. We cannot answer one of these questions without making attempts to answer the remaining two questions (Pross 2011). Pross’s analysis, however, could be extended if we consider that astrobiology is not only interested in the question, how we can find extraterrestrial life, but also in another fundamental problem, where could life spontaneously emerge. According to such an approach, astrobiology is considered as strongly related to the research dealing with the origin of life.

Astrobiology, however, makes the problem complicated. As we have no broadly accepted definition of life, exploration of organic molecules and water is still the main way of searching for extraterrestrial life forms (Chyba and Hand 2005) However, astrobiologists often postulated that living beings could be built differently than their earthly counterparts (Schulze-Makuch and Irwin 2006). This hypothesis has been recently extended to the so-called alternative forms of life which probably exist on our planet. Carol Cleland and Shelley Copley claim that our methods of detecting and examining microbes are ‘blind’ when looking for different kinds of biochemistry. So even on Earth, we can expect organisms with different genetic codes, using more amino acids than all the known forms of life or using amino acids with different chirality or maybe even more distinct from ‘standard’ life forms (Cleland and Copley 2005). Observation of GFAJ-1 bacteria from Mono Lake, which are capable of using arsenic instead of phosphorus, seems to be the first step to confirm this hypothesis (Wolfe-Simon et al. 2010). (This case, of course, should be very carefully interpreted.) However, if this assumption is true, the question about artificial creation and the natural emergence of life can be more complicated and challenging. The question whether it is possible to create a single theory of the origin of life is especially problematic. How abstract can it be without the loss of the ability of explanation and prediction? On the one hand, we can create a definition that ‘captures’ all life-like objects, but it does not refer to the mechanisms of their emerging. On the other hand, we can construct a definition that implies a specific scenario of the origin of life, but it is very restrictive. Both solutions have their weaknesses.

Another challenge of defining life is the artificial life (further referred to as ALife). The thesis that the form of living beings can be separated and implemented in a medium other than that made of organic compounds (in other words, the thesis about the multiple realization of life) is broadly shared by theorists of strong ALife (Emmeche 1992; Boden 2000). Contrary to astrobiology, this claim is strongly supported by a lot of real artificial objects (mainly computer simulations) that manifest most of fundamental life’s properties such as reproduction, evolution, self-sustainability or purposeful-like behavior. The most popular ones are Christopher Langton’s Loops, Thomas Ray’s Tierra and Craig Reynolds’ Boids (Swan 2009). The thesis about the multiple realization of life makes all attempts to define living phenomena based on physicochemistry very complicated if not impossible. If we accept it, we have to deny the possibility of giving a single answer to the questions: how can we “build” life? and how can life spontaneously emerge? On the other hand, we can of course ignore ALife objects, e.g., due to their different ontological nature. But then we are faced with an uncomfortable dilemma, as we must explain what the nature of differences and—more importantly—why we agree with the multiple realization hypothesis according to astrobiology denying at the same time multiple realization hypothesis according to ALife. The concept of biological convergence also fully corresponds to the aforementioned thesis about the multiple realization of life (indeed, one of the multiple realization concept fathers—Jerry Fodor appealed exactly to this part of biological knowledge (Block and Fodor 1972)).

## Evolutionary definitions

The above analysis demonstrates that there are a lot of problems connected with the definition of life and that one issue is especially important. It is the conflict between the functional and the physico-chemical approaches. Some authors suppose that this state (lack of consensus) is normal and cannot be changed. Edouard Machery claims that researchers from different branches of biology are interested in developing different definitions of life. It results from the discrepancy between various disciplines. Astrobiologists try to formulate a biochemical definition due to the practical facility of use, but ALife aspires to a functional definition because it is an attempt, for example, to synthesize real life in a non-organic medium (2011). Machery also believes that there is no instance of judgment which is able to choose between different definitions (2011). Similar skeptical arguments are proposed by Cleland and Chyba. According to their view, creation of a universal definition of life is impossible, because we do not have any kind of mature biological theory. The aforementioned authors compare our modern attempts of defining life to the attempts of defining water after molecular chemistry (Cleland and Chyba 2002). So, although we are able to generate a lot of definitions, there is no reason to consider one of them as correct.

The above-mentioned reflections are surprising for many reasons. First of all, they seem to ignore the synthetic theory of evolution—the central, fundamental and most integrative theory of modern biology. The synthetic theory of evolution can be used, at least in some degree, to evaluate different propositions of life definitions. Consequently, there is no wonder that the synthetic theory of evolution has been often used to define life. The most famous attempt is the so-called Darwinian or standard definition of life, created by G.F. Joyce. According to that definition ‘life is a self-sustaining chemical system capable to undergoing Darwinian evolution’ (Luisi 1998; Cleland and Chyba 2002; Ruiz-Mirazo et al. 2004). This definition is often said to be one of the most theoretically mature attempts to define life and has even reached the status of NASA’s working definition of life (Luisi 1998; Ruiz-Mirazo et al. 2004).

Before Joyce’s definition, the evolutionary definition of life was put forward by many theorists including John Maynard Smith. In the first chapter of his book *The Problems of Biology,* he analyzed the conditions of evolution. According to the author, there are three fundamental prerequisites of evolution: variability, reproduction and inheritance. A set of elements (in other words, a population of individuals) will be changed in time (evolve), if its elements share these three properties and under the condition that at least a part of variability influences the chance of their survival and reproduction (Maynard Smith 1986). In other words, a living thing is an object which belongs to a set of elements characterized by variability, reproduction and inheritance. I propose to call this kind of definition a purely evolutionary definition. There are many other examples of evolutionary definitions. One of them is created by Kepa Ruiz-Mirazo, Juli Peretó and Alvaro Moreno and it defines a living being as an ‘autonomous system with open-ended evolutionary capacities’ (2004). However, according to this view, evolution can be interpreted more widely than in accordance with the standard or the purely evolutionary definition. With some objections also, the cybernetic definition of life by Bernard Korzeniewski should be considered as one of the evolutionary definitions’ family. The author of this conception reinterprets standard evolutionary requirements for life in cybernetic terms (Korzeniewski 2001, 2005). However, some of his conclusions are contrary to the genetic interpretation of evolution (selfish gene hypothesis) (Korzeniewski 2005). Notwithstanding this fact, it seems to be a very original conception with great emphasis on formal and methodological aspects of life’s definition.

The advantages of the evolutionary approach to life definition are difficult to overestimate. Firstly, the definition is grounded in the framework of the proven biological theory; it allows to reject a lot of skeptical arguments based on linguistic reasons (lack of transparency, ambiguity and other linguistic problems are often used against the possibility of life definition—see works of Oliver and Perry (2006) or Cleland and Chyba (2002), for example). This theoretical frame also shows relations between the terms used to build the definition, so the said definition is not a simple list of properties (El-Hani 2008).

Second, evolutionary definitions (with some exceptions such as Ruiz-Mirazo’s and co-authors’ propositions) refer to a specific kind of biogenesis models. I do not mean any specific model, but rather a family of models in which it is assumed that replication emerged before self-maintenance and cellularity (Luisi 1998). Obviously, a pure evolutionary definition does not specify if it is a variant of Eigen’s hypercycle theory, a variant of RNA world hypothesis or something similar, nevertheless it provides a solid frame of research. This is a very important fact. According to this view, the relation between defining life and the scenarios of its origin is necessary to avoid making the problem of defining life a semantic problem only (Luisi 1998; Ruiz-Mirazo et al. 2004). In other words, the reciprocal impact of the definitions and the origin of life scenarios add empirical sense to the definition.

Furthermore, all evolutionary definitions have a tendency to be highly abstract, but they are not useless due to the possibility of ‘filling up’ their more concrete empirical content. We can demonstrate, for example, what mechanisms are responsible for inheritance in case of the known organisms. Moreover, we are able to predicate (certainly in some degree) what features a hypothetical extraterrestrial or alternative life must have (Maynard Smith 1986). An abstract character makes this kind of definition attractive for the non-classic disciplines of biology such as astrobiology or artificial life. Thus, the thesis about multiple realization of life is saved.

## ‘Mule’s problem’

The aforementioned advantages of evolutionary definitions of life have been strongly criticized. One of the arguments is considered especially deadly for the Darwinian approach. It refers to the simple consideration that organisms without reproductive capacity are inanimate objects in the light of the above definition. It is, of course, strongly counter-intuitive.

There are a lot of versions and variations of this argument. In the most typical version, the sterile animal is a mule—a hybrid of a horse and a donkey (Cleland and Chyba 2002; Korzeniewski 2005; Machery 2011; see also Oliver and Perry (2006)). This is also the strongest version. For this reason, I will call this argument ‘mule’s problem’. But in some variations, other objects are considered, for example, single members of any given species and even humans who do not reproduce by choice (Koshland 2002; Korzeniewski 2005). This argumentation is not always considered in the context of evolutionary definitions as the ‘mule’s problem’ is more universal and is connected with all kinds of life definitions which hold that reproduction is a fundamental property of life. But this line of argumentation is always the same—it is reductio ad absurdum. Such definitions claim that the mule is both an animate and an inanimate object.

The antagonists’ argumentation has never been precisely demonstrated, probably due to fact that the character of mule’s case is considered as problematic ‘per se’. However, some authors try to challenge this argumentation. Bernard Korzeniewski, for example, claims that

‘The reasoning concerning the non-reproduction people applies equally well to other biological individuals that do not reproduce, for example to sterile inter-species hybrids such as mule. Again, they are defective living individuals. They are alive in sense that they perform all biological function but reproduction (···), like a defective TV set performs many electronic functions but display image. However, they are completely nonsensical from the purely biological point of view’ (Korzeniewski 2005).

The line of defense mentioned above, however, has a lot of weak sides. One of its main faults is that it leads to a conclusion that evolutionary definitions cannot be used in the exact and exceptionless manner.

Another line of argumentation is proposed by Tibor Gánti—the author of chemoton theory. It must be highlighted that this is not an evolutionary theory, because the self-maintenance abilities of living units are considered more fundamental than the ability to evolve. However, Gánti’s proposition is quite often considered as a solution to the ‘mule’s problem’ (and similar problems).

Gánti distinguishes two groups of life criteria: real or absolute criteria of life and potential criteria of life. The first kind includes inherent unity, metabolism, inherent stability, information carrying subsystem and a program control. According to Gánti, these phenomena are necessary for a system to be considered as a living system. The living system displays these phenomena in every moment of existence (Gánti 2003). The second kind, which is more important for the present analysis, includes growth and reproduction (reproduction is considered a specific type of growth), a capability of hereditary change and evolution, and mortality. Gánti claims that these phenomena are not necessary for the existence of any single individual but are ‘indispensable to the survival of the living world’ (Gánti 2003). The author of the chemoton theory argues that this difference allows to avoid the paradox connected with sterile hybrids, too old or too young organisms to reproduce, castrated animals, etc., because the ability to reproduce is only potential (Gánti 2003). The same point of view is represented by his critics—Eörs Szathmáry and James Griesemer (Szathmáry 2003; Griesemer 2003). Eörs Szathmáry writes even that Gánti’s solution cuts off ‘the endless debate about whether (…) mule is alive or not’ (Szathmáry 2003). Szathmáry claims that Gánti’s idea allows to clearly demonstrate that a set of objects able to evolve and a set of living units are two different sets. They have not only a common part (‘normal’ organisms), but also separated parts (viruses, replicating programs on one hand and mules and old animals on the other, for example) (Szathmáry 2003).

But, is it really a satisfying solution? I could agree with Szathmáry’s view of two overlapping sets, because this can be easily adjusted to an evolutionary definition, especially to the NASA’s working version. (This definition also excludes only self-sustaining and only evolving objects from the set of living entities.) But I deny that Gánti’s solution can be uncritically accepted. In my opinion, the reason for distinguishing the two criteria types is not clear enough. The claim that potential criteria are necessary for survival of life is wrong from an evolutionary point of view. There is no known evolutionary mechanism whose goal is to save the life of biosphere. This hypothetical mechanism should have some kind of long-term anticipation ability, which grants access to the knowledge of the future environmental conditions. Evolution, however, is not able to predict the future. Individuals that are better adapted to the environment right now have a better chance to reproduce, unlike these who could be able to better reproduce in the hypothetical future conditions. The conclusion is that although Gánti correctly recognized that sterile animals are living individuals, he did not give us any solid reasons for accepting this point of view.

Below, I will present how the evolutionary definition can deal with this argument without a resort to exceptions (like in the Korzeniewski’s propositions) and without appealing to the unknown mechanisms of evolution (like in Gánti’s point of view). To fulfill this goal, we must first consider the ‘mule’s problem’ argumentation in detail:

1. Darwinian definition claims that living is that, what is capable of undergoing evolution through natural selection.
2. One of the conditions of evolution is reproduction.
3. So mule is unable to reproduce.
4. Therefore, mule is an inanimate object.
5. The conclusion is absurd, so the definition must be wrong.

If we want to prove that the above reasoning is false, we must reconstruct a weaker version of this argument. It will be an artificially prepared argument but not essentially different from those dealing with isolated animals or humans who do not want to reproduce. Let us consider an animal which died before reproduction took place. Points (1) and (2) do not change.

(3) The animal did not reproduce.

(4) The animal was an inanimate object.

(5) The conclusion is absurd, so the definition must be wrong.

Now we clearly see the mistake hidden in the argumentation. If the natural selection requires variability of survival and reproduction, the thesis that all organisms must reproduce themselves is certainly wrong. Evolutionary definitions holding that life evolves through natural selection say something completely different. Some organisms (in certain environmental conditions) will not reproduce at all or will have lower number of descendants than their competitors.

Where does the force of this argumentation come from? I suppose that it stems from the faulty formulation of the first premise (even if only implicitly). It is suggested that if something is living, it must evolve and because we usually think that ‘it’ must be an equivalent of ‘individual’, we arrive at the conclusion that an individual must be able to reproduce. But it is of course a mistake because evolution is not a property of individuals, it is a property of populations! Reproduction must be a property of objects belonging to a population, but there is no requirement that all objects in the set must manifest this property in the same degree.

How does the above analysis affect the ‘mule’s problem’? The aforementioned mule is in no way a more problematic case than the hypothetical animal mentioned above. Someone can say that a pack of mules will obviously never evolve. However, it is a wrong perspective. This paradox disappears if we consider the case from the genes point of view. The espousal of the genetic perspective does not change the conditions of evolution but instead of speaking about population we talk about gene pools. In this case, it is certainly obvious that a mule shares the same genes as its parents, which are capable of reproduction, and other relatives, which are not sterile hybrids. The gene pool containing genes of ‘our mule’ can of course evolve through natural selection. The conclusion is the following: mule is a living individual, because it is a part belonging to an evolving population. To be precise, a mule—considered as a vehicle of survival—is a living object, because of owning the genes which constitute a part of an evolving gene pool. Evolutionary definitions are collective definitions and an object cannot be accounted as a living or non-living one without a broader population perspective.

This does not mean that the population perspective is sufficient to define life. The living objects should be defined on the basis of other characteristics too. Otherwise, we face the problem of other objects generated by population (e.g., artifacts or excretions). Should they consider living entities? Of course they should not. Although they are the products of population, they do not share the above-mentioned additional features. But what are these additional properties? This is the matter requiring subtle investigations. However, probably the most important of them is self-maintenance (metabolism). But the relations between metabolism and evolution are not clear enough. The result is that we are unable to make a universal definition of life, although we can propose a working version. In my opinion, it could have the following form: ‘Life (a living individual) is a self-sustaining object belonging to a set of elements capable of undergoing Darwinian evolution’. As we can see, this definition is very similar to the Joyce’s conception. However, contrary to Joyce, I consider self-sustaining as an ability of any single individual, not of the whole system. According to this view, the word ‘life’ as a synonym of a ‘living individual’ should refer only to the members of population. Any elements of the members or any systems more complex than population should be called ‘alive’.

Now we can also see the nature of the above-mentioned Gánti’s conception. His solution refers to a non-collective view upon life. Therefore, Gánti has to suppose that reproduction is one of the potential criteria that can be applied to avoid the ‘mule’s problem’. But this supposition makes his conception sensitive to another type of criticism. Contrary to Gánti’s solution, the evolutionary conception of life treats life as a collective phenomenon, therefore the capacity of evolving (the reproduction is one of requirements for evolution) is considered as a real and not a potential property of life. The little differences in the perception of life makes Gánti’s conception and the Darwinian definition face two different types of problems, although they can be considered quite similar (at least at some level of abstraction). But as I tried to show above, the main problem with the Darwinian definition and other evolutionary conception is rather illusive.

## Life as a collective phenomenon

Some authors accurately recognize that the Darwinian definition is a collective definition and that it is impossible to use it to evaluate the status of objects not belonging to some higher level system (population) (Luisi 1998, Ruiz-Mirazo et al. 2004). But some of them also consider this fact as a great disadvantage of a standard definition of life, e.g., Luisi claims that this feature makes this definition useless especially for astrobiology, because it is not operational. Let us suppose that we find a single object on another planet. We cannot decide if it is a living individual or not (Luisi 1998). The second problem involves observation of evolution: how long must we wait to record effects of natural selection (Luisi 1998)? Similar arguments are discussed by Cleland and Chyba (2002).

These arguments are strongly interrelated, but the second one is easier to defeat. Contrary to the popular belief, the process of natural selection can be observed (Carroll et al. 2007). The rate of change in a population is related to the duration of the individuals’ life cycle. Therefore, in the case we most expect of extraterrestrial life which is the existence of alien microorganisms, we will be able to observe the functioning of the fundamental mechanisms of evolution. Even if we cannot observe this process, e.g., due to a long life cycle of species’ members, we can establish theoretically that these organisms are capable of undergoing Darwinian evolution. As Pier Luigi Luisi suggests, we would be able to evaluate it because we can study genetic mechanisms of these life forms (Luisi 1998). Benner shares the same point of view (2010). According to him, we are able to identify the molecules which can support the evolution through natural selection (Benner 2010), since any kind of biopolymer that is able to support the Darwinian evolution must be able to change its structure (and this property is connected with the ability of changing the information that is carried by this chemical compound) without the loss of ability to replicate. Benner says that only a few known chemical structures can fulfill this requirement (2010). The comparative studies of nucleoacids (RNA and DNA) lead to the conclusion that Darwinian evolution is possible due to specific structural features of these compounds. As Benner explains,

‘the phosphates that link the nucleoside building blocks together in the DNA backbone each carry a negative charge. This makes DNA a *polyelectrolyte*, a molecule with multiple charges. The repeating backbone charge dominates the properties of the DNA molecule so much that changing one of the uncharged nucleobases (and thereby changing the information encoded by the DNA molecule) scarcely alters the physical behavior of the DNA molecule.

The repeating backbone charge helps DNA support Darwinian evolution in other ways (···). For example:

- The repeating backbone charge keeps RNA and DNA dissolved in water.
- The repeating backbone charge forces interactions between strands to occur as far from the backbone as possible, as the backbone charges from one strand repel the backbone charges from the other (···). The Watson–Crick interactions essential to the ability of DNA to replicate arise because of interstrand interactions far from the backbone.
- The repeating backbone charge keeps the DNA and RNA molecules from folding, allowing them to act as templates’ (Benner 2010).

Without entering into the details of Benner’s hypothesis, we can claim that this proposition is very earth-centric (the polyelectrolyte hypothesis presented by Benner does not assume the thesis of the multiple realization of life). As a matter of fact, this issue is correctly recognized by the author himself (Benner 2010). I therefore argue that the observational method of detection of the evolution process through natural selection is more accurate (even if it is more difficult from a technical point of view), especially if we deal with microorganisms. It is also easy to notice that the above-mentioned methods do not exclude each other. We can use them both to verify if the population of the foreseeable alien organisms is able to fulfill the requirements of the Darwinian evolution.

However, what can we say about the situation mentioned in the first more sophisticated argument? What can we do when we find a single object only? I argue that this argumentation assumes a wrong point of departure known from science fiction movies. The suppositions that astronauts or artificial probes can find highly complex organisms in any given moonscape is nonsensical due to the fact that complexity of organisms is proportional to the complexity of their ecological niche (Korzeniewski 2005). So it is rather impossible to discover any kind of an ‘alien elephant’ in an environment without other highly complex living structures. This argument also applies to the unicellular organisms. However, in the case of microbes, we can construct additional reasoning. If we consider simple organisms such as terrestrial bacteria, it is rather impossible to find a single member of species itself. If we suppose that extraterrestrial microbes are a product of Darwinian evolution, we must also suppose that they adapted to their surroundings. So are we justified to assume that they will occur in endemic numbers? We should rather suppose that they will be found in a quantity compared to the numbers of terrestrial bacteria or archaea. And they are practically always represented in large numbers even in extreme environments. For example, the psychrophilic and psychrotolerant bacteria received from sea-ice of Arctic Ocean amount to 46,180 cfu/ml, (Helmke and Weyland 2004). So I claim that the doubts about the operational character of the Darwinian definition are magnified. The arguments presented above are valid only if we suppose that they are rather of a technical than of a fundamental nature. However, let us consider the fact that if we treat all technical problems as reasons to stop research, the science will not be able to make progress.

It is of note that the above-mentioned arguments involve reasoning by analogy. For this reason, they can be fallible. But I suppose that we should assume some universality of laws, mechanisms and properties, if we discuss the alternative forms of life. Otherwise, it is completely incomprehensible, why we want to use the word ‘life’ in relation to these alternative forms. This supposition does not only apply to the problem of the quantity of extraterrestrial organisms. It also refers to the problem of the evolution pattern. I claim that it is very difficult to imagine the evolution process totally excluding the natural selection mechanisms. The Lamarkian evolution is usually considered a counter-example, but it is not correct, because the Lamarkian evolution does not deny the natural selection, but rather complements it. Let us consider the RNA organisms in the RNA world. Cleland and Chyba claim that they are problematic for the Darwinian definition (2002). But is it really true? It is not, because the RNA organisms can still undergo the evolution through natural selection, even though the Darwinian mechanisms are supported by other kind of evolution. According to Darwinian approach to defining life, there is no reason to exclude the Lamarkian-like organisms, if they are able to undergo the Darwinian process too. I suppose that the satisfying definition of life should ‘catch’ the minimal properties of life (common between all life’s forms). The additional features (e.g., inheritance of acquired properties, learning, complex behavior or consciousness) should be omitted. The same reasoning applies to ALife objects. Although some of them can exhibit non-Darwinian patterns of evolution, it is rather difficult to imagine the real-like environmental conditions without influence on the chance of reproduction of ALife objects. The conclusion is that although the Darwinian approach to defining life generally does not exclude the multiple realization of life, not all the entities created by A-lifers should be considered living individuals.

## Conclusions

I tried to demonstrate that the Darwinian and other kinds of evolutionary definitions are not as vulnerable as their critics say. ‘Mule’s problem’, allegedly so problematic, seems to be due to a confusion of the biological hierarchy levels in which objects are able to evolve (namely the populations but not the individuals). Moreover, the arguments relating to the operational character of definition fail if we consider life as a collective phenomenon. However, it should be emphasized that the Darwinian definition (or my own working definition mentioned above) does not dispel all doubts about the problem of defining life. It does not cast enough light on the problem of the relation between evolution and the second major property of life—self-maintenance. As Freeman Dyson suggests, the research of the relationship between these features, namely the evolution and self-maintenance and their mutual connection has often been neglected (Dyson 1985). Ruiz-Mirazo and his co-workers also argue that the evolutionary definition, even in its more sophisticated form of the standard life definition, does not explain the character of ‘material organization of living that would allow to beginning of a process of Darwinian evolution’ enough (Ruiz-Mirazo et al. 2004). (Detailed Gánti’s analysis of the nature of inherent self-maintenance unity should be in this context very useful, if it can be adjusted—and I think it can—to the evolutionary point of view.) Another unsolved problem is the status of ALife objects. Evolved programs should be recognized as life in the context of a ‘pure evolutionary’ definition. But even if they evolve according to the pattern of Darwinian evolution, are there really no differences between them and standard organisms? NASA’s working definition excluded ALife objects; the reason is that they are not chemical systems. However, we are faced with another question. We must explain why we cannot claim that this assumption is not arbitrary. These issues require further investigations and research.
